# Encounters between medical specialists and patients with medically unexplained physical symptoms; influences of communication on patient outcomes and use of health care: a literature overview

**DOI:** 10.1007/s40037-012-0025-0

**Published:** 2012-09-27

**Authors:** Anne Weiland, Rianne E. Van de Kraats, Annette H. Blankenstein, Jan L. C. M. Van Saase, Henk T. Van der Molen, Wichor M. Bramer, Alexandra M. Van Dulmen, Lidia R. Arends

**Affiliations:** 1 Department of Internal Medicine, Erasmus MC, University Medical Center, 2040, 3000 CA Rotterdam, the Netherlands; 2 Faculty of Social Sciences, Institute of Psychology, Erasmus University Rotterdam, Rotterdam, the Netherlands; 3 Department of Psychiatry, University Medical Center Utrecht, Utrecht, the Netherlands; 4 Department of General Practice & Elderly Care Medicine, VU University Medical Center, Amsterdam, the Netherlands; 5 Department of Internal Medicine, Erasmus MC, University Medical Center, Rotterdam, the Netherlands; 6 Faculty of Social Sciences, Institute of Psychology, Erasmus University Rotterdam, Rotterdam, the Netherlands; 7 Medical Library, Erasmus MC, University Medical Center, Rotterdam, the Netherlands; 8 Netherlands Institute for Health Services Research (NIVEL), Utrecht the Netherlands,; 9 Department of Primary and Community Care, Radboud University Nijmegen Medical Centre, Nijmegen, the Netherlands; 10 Department of Health Science, Buskerud University College, Drammen, Norway; 11 Faculty of Social Sciences, Institute of Psychology and Institute of Pedagogy, Erasmus University, Rotterdam, the Netherlands; 12 Department of Biostatistics, Erasmus MC, University Medical Center, Rotterdam, the Netherlands

**Keywords:** Medically unexplained physical symptoms, Physician-patient relations, Communication, Medical specialists, Use of Health Care

## Abstract

Medically unexplained physical symptoms (MUPS) burden patients and health services due to large quantities of consultations and medical interventions. The aim of this study is to determine which elements of communication in non-psychiatric specialist MUPS care influence health outcomes. Systematic search in PubMed, PsycINFO and Embase. Data extraction comprising study design, patient characteristics, number of patients, communication strategies, outcome measures and results. Elements of doctor-patient communication were framed according to symptoms, health anxiety, satisfaction, daily functioning and use of health care. Eight included studies. Two studies described the effect of communication on patient outcome in physical symptoms, three studies on health anxiety and patient satisfaction and one study on daily functioning. Two studies contained research on use of health care. Qualitative synthesis of findings was conducted. Communication matters in non-psychiatric MUPS specialist care. Perceiving patients’ expectations correctly enables specialists to influence patients’ cognitions, to reduce patients’ anxiety and improve patients’ satisfaction. Patients report less symptoms and health anxiety when symptoms are properly explained. Positive interaction and feedback reduces use of health care and improves coping. Development of communication skills focused on MUPS patients should be part of postgraduate education for medical specialists.

## Introduction

Communication, defined as the intentional verbal and non-verbal actions of a health professional, is generally understood to be an important component of patient care [1]. A systematic review of randomized clinical trials and descriptive studies about physician-patient communication indeed revealed a positive influence of effective communication on health outcomes [2]. When physicians have no medical explanation for persisting physical symptoms (e.g. chronic fatigue syndrome, irritable bowel syndrome (IBS), chronic pain syndrome, fibromyalgia syndrome, chronic pelvic pain, pseudo non-epileptic seizures) many patients feel that they are not being taken seriously, whereas doctors often feel unable to come to an agreement with their patients on problem definition [3]. Dissatisfaction and pressure on the doctor-patient relationship hamper their communication. The health outcome of patients with MUPS in primary care can be influenced positively by patient-centred communication, effective reassurance, reliable patient information and a clear and positive explanation about the nature of the symptoms [4–8].

Patient-centred communication in general is incorporated in Dutch undergraduate medical education. MUPS-focused communication skills training is available in postgraduate education for GPs and trainees [9] but not for medical specialists and residents.

Since at least 40 % of physical symptoms presented in outpatient clinics of gynaecology, neurology or rheumatology remain medically unexplained [10–12], medical specialists could benefit from MUPS-focused training programmes. MUPS burden patients and health services due to large quantities of consultations and medical interventions [13]. Comorbidity, lack of clear guidelines and limited knowledge about MUPS among non-psychiatric specialists [14–16] often cause unnecessary medical interventions and unintentionally reinforce somatisation [17]. Normal test results of additional specialist investigations naturally do not reassure MUPS patients [18, 19].

In short, MUPS in specialist care is a big issue. Therefore, we want to explore what is known about effective physician-patient communication in MUPS specialist care. Are there MUPS-focused communication strategies for specialists? Does communication matter in MUPS specialist care?

### Objective

To study the questions above, our objective is: ‘Which elements of doctor-patient communication by non-psychiatric specialists in patients with MUPS influence symptoms, health anxiety, satisfaction, daily functioning and use of health care?’ These specific outcome measures were used in different types of health care research [20–24]. MUPS specialist care, being far more costly than general care, could benefit from improving these outcomes.

## Methods

### Data sources and search strategy

We conducted systematic searches in the electronic databases PubMed, Embase and PsycINFO in April 2011. Medically unexplained physical symptoms was searched in four different ways. The word ‘unexplained’ and its synonym was combined with ‘subjective symptoms’ and its synonyms, with behaviours often occurring in MUPS patients and for general complaints (such as headache) combined with factors that make it unexplained (such as chronic). This search for MUPS was combined with a search for non-psychiatric specialist or secondary care and their synonyms and with a search for interaction as a combination of synonyms for the word professional near the word patient. Table 1 shows the complete search string in Embase.

**Table 1:** Search for www.embase.com

#1	(unexplain* OR (un NEXT/1 explain*) OR (‘not’ NEXT/3 explain*)):de,ab,ti
#2	(nonspecific* OR (non NEXT/1 specific*) OR (‘not’ NEXT/3 specific*)):de,ab,ti
#3	((subjective OR Somatoform OR functional) NEXT/5 (symptom* OR disorder* OR complaint*)):de,ab,ti
#4	((frequent NEXT/1 attend*) OR (high NEXT/1 utili*) OR hypochondri*):de,ab,ti
#5	((Headache OR ‘chest pain’ OR ‘neck pain’ OR ‘pelvic pain’ OR ‘benign pain’ OR ‘back pain’ OR trauma OR ‘chemical sensitivity’ OR gastrointest* OR dyspepsia OR seizure* OR Fatigue OR dizziness OR hysteri* OR premenstrual OR ‘irritable bowel’ OR fibromyalgia) NEAR/3 (psycholog* OR psychogen* OR Psychosom* OR Psychophysiol* OR functional* OR chronic OR syndrome OR non-cardiac OR noncardiac OR Tension OR cumulative OR multiple)):de,ab,ti
#6	#1 OR #2 OR #3 OR #4 OR #5
#7	(specialis* OR specialization OR physician* OR (vocational NEXT/1 trainee*) OR intern OR interns OR resident* OR ‘secondary care’ OR hospital*):de,ab,ti
#8	((professional* OR doctor* OR physician* OR provider*) NEAR/3 patient):de,ab,ti
#9	#6 AND #7 AND #8

### Study inclusion and selection

Studies were eligible for selection if they were published in peer-reviewed journals in English, German, French or Dutch; involved an adult human population; had a publication year between January 1984, when PubMed started, and April 2011; had an empirical study design; and contained an outcome at patient level in terms of symptoms, health anxiety, satisfaction, daily functioning or the use of health care. After removing the duplicates, two authors (AW, RK) independently screened titles and abstracts to select eligible studies; selection was checked by two co-authors (AB, LA), who each revised the first selection. Full text papers were obtained of the selected studies. AW and RK independently critically appraised the full-text papers and excluded studies that did not meet the inclusion criteria. Disagreement was solved by discussion between authors (AW, RK, AB, LA).

### Data extraction and analysis

For all included studies, data extraction was undertaken comprising study design, patient characteristics, number of patients, communication aspects, and outcomes, as shown in Table 2. Meta-analysis was not feasible due to the small number of studies and variety in study design and outcome measures; therefore a qualitative synthesis of findings was conducted.

**Table 2:** Overview of included studies

Author, year^Ref **#**^	Study design	Study group	Number of patients	Outcome	Intervention/study subject	Effect
Bieber 2008 [30]	RCT	Fibromyalgia syndrome patients	83	Patient satisfaction	A shared decision-making training programme for specialists combined with an information leaflet for patients versus information leaflet only	No difference in patient satisfaction was found in the shared decision making group and the information only group
Bieber 2006 [31]	RCT	Fibromyalgia syndrome patients	67	Functioning	A shared decision-making training programme for specialists combined with an information leaflet for patients versus information leaflet only or standard care as usual	Functional capacity did not differ across the study groups. The patients of the share decision making study group improved coping with pain and being more positive
Collins 2009 [28]	Cohort study	Patients with functional gastrointestinal disorders	13	Health anxiety Use of health care	Concordance between specialists’ understanding of patients reported symptoms and their actual needs	Underestimating patients’ expectations and symptoms maintained health anxiety and was likely to lead to more use of health care
Van Dulmen 1995 [27]	Cohort study	Patients with functional abdominal pain	110	Patient satisfaction Health anxiety	Correct perceptions of patients’ attributions and having the same doctor	Reduced health anxiety (*p* = 0.01) and improved satisfaction by consulting the same doctor (*p* = 0.02)
Hall-Patch 2010 [25]	Cohort study	Patients with pseudo neurological epileptic seizures	50	Course of symptoms	A patient information leaflet and a communication protocol for neurologists to explain the psychological nature of the seizures	Reduced frequency of seizures
Owens 1995 [32]	Cohort study	Patients with irritable bowel syndrome	112	Use of health care	Physician-patient relationship on use of health care	Reduced number of return visits for IBS-related symptoms
Petrie 2007 [26]	RCT	Patients with nonspecific chest pain	92	Health anxiety Course of symptoms	Providing information about normal test results before testing	The number of patients still reporting chest pain after 1 month decreased significantly (*p* < 0.001). Addressing patients’ attributions by information about normal test results prior to testing diminished health anxiety
Stones 2006 [29]	Cohort study	Women with chronic pelvic pain	100	Patient satisfaction	Doctors affect, appropriateness of information and ability to meet patients expectations	Initial consultation influenced further care experiences. Doctors affect, appropriate information and meeting patients’ expectations enhanced patient satisfaction

## Results

### Selection of studies

**Fig. 1:**
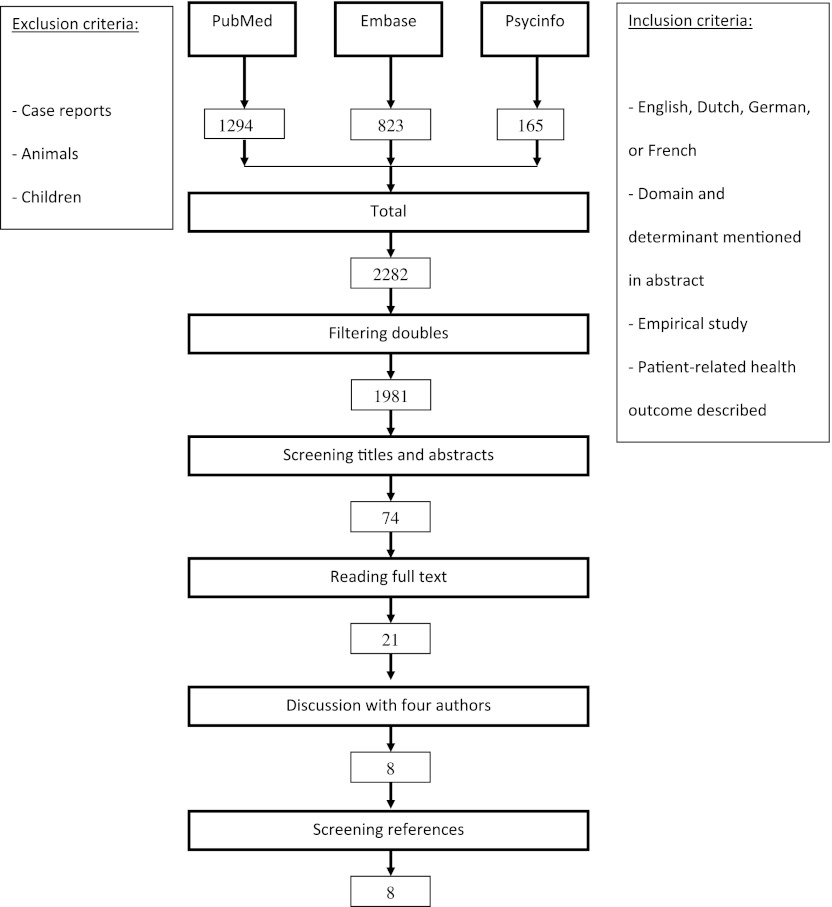
Flow chart

The combined search resulted in 1981 articles. After screening titles and abstracts, 74 articles met the inclusion criteria and were retrieved for further assessment. Two authors (AW, RK) reviewed these full-text articles and selected 21 articles according to inclusion and exclusion criteria. Discussion with four authors (AW, RK, AB, LA) reduced the number to eight eligible studies. Thirteen articles were excluded because they lacked outcomes fitting our study question. Of the selected articles, a thorough search of related articles, references and citing articles was performed. This yielded no extra article for inclusion. Figure 1 presents the flowchart of the systematic search.

### Synthesis of findings

The included articles discuss different types of MUPS patients, and describe different elements of communication strategies used by medical specialists that may have an impact on health outcomes and use of health care. We framed and summarised these elements of doctor-patient communication according to the outcomes defined in our study question: symptoms, health anxiety, satisfaction, daily functioning and use of health care.

### Symptoms

In the study by Hall-Patch et al. [25] most patients with psychogenic non-epileptic seizures (PNES) were initially diagnosed as having epilepsy and had been treated with antiepileptics for several years. Participants received the diagnosis of PNES on average 5.2 years after seizure manifestation. The study was carried out to assess the acceptability and effectiveness of a patient information leaflet and a communication protocol for neurologists to explain the psychological nature of the seizures to their patients; 44 patients positively evaluated the information leaflet. The frequency of the seizures between diagnosis and follow-up after 3 months was reduced by more than 50 % in 63 % of the patients; 14 % of the patients were seizure-free after 3 months.

Petrie et al. [26] investigated whether providing information about normal findings prior to a diagnostic test improves patients’ reassurance and reduces health anxiety. They studied 92 patients with chest pain who were referred for a diagnostic exercise stress test. These patients were divided into a group of 30 patients receiving a pamphlet explaining the function and meaning of normal test results; a group of 34 patients receiving the pamphlet and a brief discussion about the meaning of normal test results and a control group of 28 patients receiving standard information. The number of patients still reporting chest pain after 1 month decreased significantly in the discussion group (*p* < 0.001) and pamphlet group (*p* = 0.005) but not in the control group (*p* = 0.09). Another finding was that fewer patients in the discussion group were taking cardiac drugs after 1 month. In conclusion, explaining the nature of MUPS with an information leaflet, a core points crib sheet for specialists and a brief discussion about the meaning of normal test results prior to testing reduces symptoms in patients.

### Health anxiety

The study by Petrie et al. [26], mentioned above showed that the mean levels of reassurance in patients with chest pain after testing and feedback from the doctor were significantly higher in the discussion group [*M* = 42.0, 95 % confidence interval (CI) 39.7–44.2] than in the pamphlet group (*M* = 39.2, 95 % CI 36.1–42.3) and control group (*M* = 35.8, 95 % CI 31.6–39.9). This difference was maintained after 1 month. So, addressing patients attributions and providing patients with information about normal test results before testing can improve reassurance and thus diminish health anxiety.

Van Dulmen et al. [27] explored changes in complaint-related cognitions and anxiety of 110 patients with IBS during a series of consultations in an outpatient clinic of internal medicine. They found that anxiety (*p* = 0.01), fear of cancer (*p* < 0.001), somatic attributions (*p* < 0.001) and catastrophising cognitions (*p* = 0.008) diminished significantly between the first and last consultation of patients with IBS. Aspects of communication that accounted for the measured effects were doctors’ correct perceptions of patients’ attributions and having the same doctor throughout the consultations.

Collins et al. [28] studied concordance between 13 eligible patients with functional gastrointestinal disorders (FGID) and doctors (11 gastroenterologists and 13 GPs). They investigated patients’ needs and expectations at initial consultations and whether their specialists and GPs recognised these patient perceptions. Gastroenterologists underestimated patients’ reported number of symptoms (82 %), pain (48 %), and interference with daily functioning (41 %). Views on the best treatment options diverged: patients preferred operation (41 %) or diet (31 %), whereas the specialists were focused on symptom control by medication (41 %) or managing worry (28 %). A persisting expectation of finding a specific cause and cure was present in these patients. Only one out of 13 patients acknowledged the diagnosis FGID at follow-up. So, underestimating patients’ expectations and symptoms does not reassure patients and maintains existing health anxiety.

### Patient satisfaction

Van Dulmen et al. [27] found that patients whose anxiety diminished (*N* = 59) were more satisfied with the visit to the doctor than patients whose anxiety did not diminish (*p* = 0.02). Patients consulting the same doctor throughout the consultations were more satisfied with the consultations than patients who visited different doctors (*p* = 0.05).

The study by Stones et al. [29] aimed to identify the three dimensions of patient satisfaction (affect, cognition and expectation) through which initial consultations were subsequently recalled at follow-up in 100 gynaecology patients with chronic pelvic pain (CPP). These authors demonstrated that doctors’ affect, appropriateness of information and the ability to meet patients’ expectations are strong influences on experiences of care. These three elements of patient satisfaction were interrelated and influenced the experiences of care. Building a good relationship in the first hospital visit improves the understanding of the diagnosis and makes a positive coping of the patient more likely.

Bieber et al. [30, 31] assessed whether shared decision-making improves the quality of physician-patient interaction from the perspective of the patient in 85 patients with fibromyalgia syndrome. They measured patient satisfaction with the decisions and did not find significant group differences. Decisional conflicts and satisfaction with decisions were similar in the study groups.

### Daily functioning

Bieber et al. [30, 31] found that fibromyalgia syndrome patients benefit from a shared decision-making communication training programme for physicians combined with an information package for patients. During the training, doctors learned to consider their patients’ individual needs and to meet their patients’ expectations. These elements accounted for a better physician-patient interaction. Qualitative assessment revealed a dramatic difference: at 1-year follow-up more patients in the shared decision-making group (62 %) than in the care as usual group (28 %) mentioned that their coping with pain had improved. Patients from the shared decision group adopted a more positive view when thinking of the future with their illness than patients from the care as usual group.

### Use of health care

Collins et al. [28] suggest that failure of patients to acknowledge their diagnosis of FGID might underpin recurrent consultations and possibly leads to unnecessary use of health care. Patients who believe that their symptoms are not adequately explained are not able to accept the diagnosis. Collins et al. also found that when patients seek specialist consultation, the reason for the visit often remains unclear to the specialist. Possible reasons found are the need of diagnosing the cause of symptoms and the initiation or the readjustment of treatment. Effective consultation with MUPS patients starts with exploring the reason why the patient visits the doctor.

Owens et al. [32] found that a strong physician-patient interaction may be related to a reduced number of return visits for patients with IBS. Comparison of the strongest and weakest interaction groups (1.8 and 4.9 hospitalizations, respectively; *p* < 0.05) indicated that positive interaction was associated with fewer hospitalizations. However, the authors found no association between strength of the physician-patient interaction and number of surgeries. Notation in medical records of the patient’s psychosocial history (*p* < 0.01) about precipitating factors causing the patient to seek medical help (*p* < 0.01) and notation of discussions with the patient (*p* < 0.02) were associated with fewer follow-up visits for IBS-related symptoms.

## Discussion

### Main findings

This review demonstrates that the research on specialist communication with MUPS patients and its effect on patient outcomes and use of health care is limited. We did not restrict our search to RCTs and CCTs. Despite having broad inclusion criteria we only found 8 studies describing different outcomes and aspects of communication:

1. Perceiving patients’ expectations correctly enables specialists to influence patients’ cognitions, reduces patients’ anxiety, and improves patient satisfaction [27].
2. Explaining the nature of MUPS with an information leaflet and a core points crib sheet for specialists reduces health anxiety and symptoms in patients [25].
3. Providing patients with information about normal test results prior to investigation helps to reassure patients [26].
4. Positive doctor–patient interaction [28, 29] and positive feedback from the doctor contributes to reduced use of health care [32] and better coping with complaints in the long term [30, 31].

Incorporating these four elements in a vocational and postgraduate MUPS-focused communication skills training for specialists could improve MUPS specialist care and support specialists in their consultations with MUPS patients.

### Comparison with the literature

We found that proper explanation and showing an effect in communication with MUPS patients in specialist care improve patient outcomes and reduce the use of health care. Specialists trained in shared decision-making [30, 31] and in communicating the diagnosis MUPS to patients [27] influenced health outcomes positively. These elements are also important in general practice and in patients with minor ailments. Blankenstein et al. [33] found that trained GPs were able to apply cognitive-behavioural techniques to patients with MUPS during normal consultation hours. At follow-up subjective health was increased, use of health care and sick-leave were decreased [26]. Fassaert et al. [5] studied positive communication strategies during 524 videotaped consultations in general practice with patients with minor ailments related to medication adherence, consultation frequency, functional health status and state anxiety. Results show that, to some extent, it seems helpful when GPs are at the same time clear and optimistic about the nature and course of minor ailments. Results of this study indicate that it is important for physicians to pay attention to the patients’ mood. Thomas studied 200 patients in general practice who presented symptoms without abnormal physical signs and in whom no definite diagnosis was made. Patients who received a positive consultation from their GP for their symptoms were more likely to improve than those who received no explanation [8]. Sometimes MUPS patients are referred frequently to secondary care even after having received multiple specialist opinions that their symptoms were medically unexplained [34]. Referring MUPS patients to hospital clinics repeatedly is not the best way to address their needs [35]. These patients are unlikely to benefit from repeated referrals to specialist services that are designed to find or exclude disease rather than to deal with symptoms [36]. Positive communication between specialists and GPs is required to reduce unnecessary medical interventions, use of health care and aggravation of symptoms, and improves care for MUPS patients by sharing knowledge and stepped care [37].

### Strengths and limitations of this review

This review is the first paper to give an overview on the knowledge of doctor-patient MUPS-focused communication in specialist care. Although the selected studies contain a limited variety of MUPS, patient characteristics and aetiological mechanisms appear to be quite similar for different MUPS [38]. Therefore our results can probably be transferred to MUPS patients in general. From all selected studies, only three described explicit communication programmes for specialists [25, 26, 31].This indicates the low priority in specialist care for MUPS-focused communication. Enhancement of knowledge and communication skills might improve specialist care for MUPS patients [39, 40]. Methodological and clinical variety of the studies and small number of (quantitative) studies made pooling of results of the different studies not useful.

## Conclusion

This review shows that communication matters in specialist care. Perceiving patients’ expectations correctly enables specialists to influence patients’ cognitions, reduce patients’ anxiety and improve patients’ satisfaction. Providing patients with information helps them to feel reassured. Patients report less symptoms and health anxiety when they get a proper explanation of their symptoms. Positive doctor-patient interaction and positive feedback from the doctor reduces the use of health care and improves coping with complaints on the long term. These elements should be integrated in postgraduate education for specialists.

### Recommendations for research and post-graduate education

First, we recommend further research on communication with MUPS patients in non-psychiatric specialist care and related health outcomes. Second, we recommend research on postgraduate education in specialist care for MUPS patients to enhance communication skills for specialists that contribute to the quality of specialist care for MUPS patients.

## Essentials

- Explaining the nature of MUPS with an information leaflet and a core points crib sheet for specialists reduces health anxiety and symptoms in patients.
- Perceiving patients’ expectations correctly enables specialists to influence patients’ cognitions and reduces patients’ anxiety and improves satisfaction.
- Providing patients with information about normal test results prior to investigation helps to reassure patients.
- Positive doctor-patient interaction and positive feedback from the doctor contributes to reduced use of health care and better coping in the long term with complaints.
